# Impact of health literacy and general self‐efficacy on surgical outcomes 2 years after bariatric surgery

**DOI:** 10.1111/cob.70009

**Published:** 2025-03-10

**Authors:** Maria Jaensson, Karuna Dahlberg, Yang Cao, Anders Thorell, Johanna Österberg, Ulrica Nilsson, Erik Stenberg

**Affiliations:** ^1^ Faculty of Medicine and Health, School of Health Sciences Örebro University Örebro Sweden; ^2^ Clinical Epidemiology and Biostatistics, School of Medical Sciences, Faculty of Medicine and Health Örebro University Örebro Sweden; ^3^ Unit of Integrative Epidemiology Institute of Environmental Medicine, Karolinska Institutet Stockholm Sweden; ^4^ Department of Clinical Science, Danderyd Hospital Karolinska Institutet Stockholm Sweden; ^5^ Department of Surgery and Anesthesiology Ersta Hospital Stockholm Sweden; ^6^ Department of Surgery Mora Hospital Mora Sweden; ^7^ Department of Clinical Science and Education Södersjukhuset, Karolinska Institute Stockholm Sweden; ^8^ Department of Neurobiology, Care Sciences, and Society Karolinska Institute Stockholm Sweden; ^9^ Department of Surgery, Faculty of Medicine and Health Örebro University Örebro Sweden

**Keywords:** bariatric surgery, health literacy, self‐efficacy

## Abstract

After bariatric surgery, adherence to lifestyle recommendations is crucial. Health literacy and self‐efficacy may impact recovery after surgery. In this multicentre study performed in three hospitals in Sweden, we evaluated any relation between preoperative health literacy and general self‐efficacy on the one side and weight loss, health‐related quality of life, length of stay, and complications up to 2 years after bariatric surgery on the other. Of 686 included patients, 56% (*n* = 382) had limited functional health literacy, 42% (*n* = 278) had limited communicative and critical health literacy, and 40% (*n* = 266) reported low general self‐efficacy. Preoperative functional, communicative and critical health literacy, and general self‐efficacy were not associated with the degree of weight loss at 1 or 2 years after surgery. However, limited health literacy and low general self‐efficacy scores were associated with both reduced quality of life and obesity‐related problems postoperatively. Further, a higher proportion of those with inadequate health literacy had a prolonged length of stay. Although patients with limited health literacy and self‐efficacy may experience similar maximum weight loss after bariatric surgery as other patients, they still might have reduced health‐related quality of life in terms of obesity‐related problems. Increased awareness of this association as well as patient‐centered support before and after bariatric surgery may be of benefit.

**What is already known about this subject**

- After bariatric surgery, patients need to make significant lifestyle changes.
- Currently, there is a lack of knowledge about how levels of health literacy and general self‐efficacy affect postoperative recovery.

**What this study adds**

- Health literacy or self‐efficacy was not associated with the degree of weight loss.
- Limited health literacy and low general self‐efficacy were associated with a decreased quality of life.
- Limited health literacy was associated with prolonged length of hospital stay.

## 1 INTRODUCTION

Obesity is associated with a number of cardiovascular and metabolic comorbidities as well as reduced health‐related quality of life (HRQoL).^1^ Currently, bariatric surgery is the most effective treatment in terms of marked and sustained weight loss and improvements in metabolic and other comorbidities. After laparoscopic Roux‐en‐Y gastric bypass (LRYGB) or sleeve gastrectomy (LSG), an average loss of 28%–31% of the total weight can be expected.^2^ Reaching at least 10% total weight loss (TWL) has been reported to be associated with a reduced incidence of cardiometabolic events, but a small proportion of patients do not reach this target and a further proportion may regain weight over time.^3^, ^4^ Research has also shown an association between socioeconomic factors and the degree of weight loss, as well as the number of patients who fail to achieve sufficient weight loss.^5^

After bariatric surgery, adherence to recommendations for exercise and dietary intake is crucial.^6^ For some patients, this can be difficult. A barrier in achieving adequate weight loss can be the person's level of health literacy.^7^ “Health literacy” is defined as the person's ability to access, understand, and apply information for informed decision‐making about his or her health.^8^ Research reporting the association between limited health literacy and worse health outcomes is consistent,^9^, ^10^ but research is limited in surgical populations.^11^ Some smaller studies have suggested that limited health literacy may be associated with reduced maximum weight loss after bariatric surgery,^12^, ^13^ but conflicting results exist.^14^, ^15^ In one study, health literacy was found to be more important during the weight maintenance phase,^14^ than during the early postoperative weight loss phase.

Another factor that plays a role in lifestyle changes is the person's self‐efficacy.^16^ “Self‐efficacy” refers to a person's beliefs in his or her capacity to organise and execute actions to achieve certain goals.^16^ Previous small studies show that general self‐efficacy improves over time after surgery.^17^, ^18^ The effect of bariatric surgery on HRQoL is associated with postoperative weight loss, but it also depends on patient preoperative characteristics and socioeconomic status.^19^

To date, there is limited research examining health literacy and general self‐efficacy in patients undergoing bariatric surgery, which may both have an impact on a person's recovery after bariatric surgery. Therefore, the aims were

1. to evaluate potential associations between preoperative health literacy and general self‐efficacy on the one hand and postoperative weight loss and HRQoL up to 2 years after bariatric surgery on the other;
2. to analyse preoperative health literacy and general self‐efficacy and potential associations with postoperative length of stay, readmission, and postoperative complications during the first 30 days after bariatric surgery.

## 2 METHODS

This study was part of a prospective, longitudinal mixed‐methods study conducted at three hospitals in Sweden.

### 2.1 Inclusion and exclusion

Adults (≥18 years) scheduled for primary LRYGB or LSG and able to read and understand written and spoken Swedish were considered for inclusion, which includes more than 85% of patients undergoing bariatric surgery in Sweden.^20^, ^21^ Verbal and written information was provided, and patients were included in the study after giving their written informed consent.

### 2.2 Data collection

Before surgery, patients completed three questionnaires including the Swedish scales for functional health literacy,^22^ communicative and critical health literacy,^23^ and general self‐efficacy.^24^ In addition, data on all patients was registered in the Scandinavian Obesity Surgery Registry (SOReg). This is a Scandinavian (Swedish and Norwegian) quality and research registry including baseline data, information concerning the operation, and postoperative follow‐up and health‐related quality of life on day 30, and at 1, 2, 5, and 10 years after surgery. The registry is continuously validated and has been shown to have very high completeness and validity of data.^25^ The perioperative care at all centres was, in general, highly adherent to current Enhanced Recovery after Surgery guidelines for perioperative care, including early mobilization and commencement of oral intake. All patients had a weight‐reducing, low‐energy diet, 800–1200 kcal/day, for 2–4 weeks before surgery.^6^ Baseline data in SOReg were recorded before starting the preoperative weight‐reducing diet.

The surgical method for LRYGB is well established and standardized in Sweden. The procedure in all patients undergoing LRYGB in this study was antecolic antegastric Roux‐en‐Y gastric bypass with a small gastric pouch and a gastrojejunostomy created using linear staplers with suturing of the remaining defect. The jejunojejunostomy was created using a single or double stapling technique at the discretion of the surgeon. The mesenteric defects were closed with running non‐absorbable sutures or non‐resorbable clips, according to local routines. The LSG was created using a 35 French Bougie, starting the resection ≤5 cm from the pylorus and ending it 1 cm from the angle of His. Over‐sewing or buttressing of the staple line was optional.

Presence of comorbidity was defined as an obesity‐related condition requiring pharmacological treatment, nocturnal continuous positive airway pressure treatment (CPAP), or nocturnal bi‐level positive airway pressure treatment (BiPAP).

### 2.3 Study measures

#### 2.3.1 The Functional Health Literacy scale

Functional health literacy involves basic skills that focus on understanding information.^26^ The Swedish Functional Health Literacy scale consists of five items rated on a 5‐point Likert scale (1 = never to 5 = always), with lower scores indicating sufficient health literacy.^22^ The scale has been translated into Swedish, and psychometric tests in a Swedish population undergoing bariatric surgery have shown satisfactory reliability and validity.^27^ Based on the user's manual, functional health literacy was categorized into inadequate, problematic, and sufficient health literacy.^28^

#### 2.3.2 The Communicative and Critical Health Literacy scale

Communicative and critical health literacy involves more complex abilities (namely, to extract and analyse information).^26^ The Swedish Communicative and Critical Health Literacy scale consists of five items rated on a 5‐point Likert scale (1 = strongly disagree to 5 = strongly agree), with higher scores indicating a sufficient health literacy.^23^ The scale has been translated to Swedish and psychometric tests in a Swedish population undergoing bariatric surgery have shown satisfactory reliability and validity.^29^ Based on the user's manual, communicative and critical health literacy were categorized into inadequate, problematic, and sufficient health literacy.^28^

#### 2.3.3 The General Self‐Efficacy scale

This scale consists of 10 items rated on a 4‐point Likert scale. Each item is answered by one of the following responses: not at all true, hardly true, moderately true, exactly true. Possible sum scores range from 10 to 40; lower scores indicate lower self‐efficacy.^24^ The scale has been translated into different languages^24^ and psychometric tests in a Swedish population undergoing bariatric surgery have shown satisfactory reliability and validity.^29^ Based on the user's manual, general self‐efficacy sum scores were dichotomized into low self‐efficacy ≤30 and high self‐efficacy ≥31.^30^

### 2.4 Outcomes

The main outcomes for this 2‐year follow‐up study were weight reduction and HRQoL. Weight reduction was measured at 1 and 2 years after surgery and presented as loss of body mass index (BMI loss), excess BMI loss (EBMIL = (baseline BMI − follow‐up BMI)/(baseline BMI − 25)), and TWL = (baseline weight − follow‐up weight)/(baseline weight) × 100 (%).

HRQoL was evaluated as obesity‐related problems using the Obesity Related‐Problems (OP) scale and the RAND‐36.

The Obesity‐Related Problems Scale is designed to measure the impact of obesity on health‐related quality of life (HRQoL).^31^ It consists of eight items about common obesity‐related problems. The scores are summed, the total score ranging from 0 to 100, with lower scores indicating better psychosocial functioning. The scale has undergone psychometric evaluation in a Swedish population, demonstrating satisfactory reliability and validity.^31^

The RAND‐36^32^, ^33^ has 36 items grouped into eight multi‐item scales that measure physical function, role function, bodily pain, general health, vitality, social functioning, emotional role, and mental health. The scales are scored from 0 to 100, with a higher score indicating better health status. The non‐normative physical component summary and mental component summary scores were calculated according to Andersen et al.^34^ The instrument has undergone psychometric evaluation in a Swedish population, demonstrating satisfactory reliability and validity.^32^, ^33^

Postoperative complications were graded according to the Clavien‐Dindo classification of postoperative complications, with a complication graded as ≥3b being a serious postoperative complication.^35^

### 2.5 Statistics

The sample size calculation was based on the assumption of a clinically relevant reduction in EBMIL from the average weight loss after LRYGB in Sweden of 82%, as reported in the SOReg, to 75% for persons with limited health literacy.^20^ To detect such a difference with a power of 80% at the 5% significance level, a sample size of 624 patients would be needed, resulting in a study group of 700 patients to allow for 10% attrition.^20^

Baseline data is presented as mean ± standard deviation (SD) for continuous variables and frequency (percentage) for categorical variables.

Missing values were imputed using the multivariate imputation by chained equations method, where multivariate linear regression was applied to continuous variables, logistic regression to binary variables, and ordered logistic regression to ordered variables.^36^ A total of five imputed datasets were created. In each of these, dichotomous outcomes were analysed using log‐binomial regression models, while continuous outcomes were examined using quantile regression models to assess group differences. The final results were compiled by aggregating results from the five datasets, following Rubin's rules.^37^ The main analyses were adjusted for baseline BMI, age, sex, and surgical method. A two‐sided *p*‐value <.05 was considered statistically significant. Data management and statistical analyses were performed using IBM SPSS version 29 (IBM, Armonk, NY, USA) and Stata 18.0 (StataCorp, College Station, TX, USA).

### 2.6 Ethics

The study was approved by the regional ethics board in Uppsala (ref:2018/256) and followed the principles of the 1964 Helsinki declaration and its later amendments.

## 3 RESULTS

From 15 October 2018 until 3 November 2020, 686 patients who underwent a primary bariatric surgical procedure and met the inclusion criteria were included in this study. Two patients died during the first 2 years post‐surgery, one from cancer and one from a cardiovascular event. Follow‐up data at day 30 was registered for 681 patients (99.3%), at 1 year after surgery for 651 patients (94.9%), and at 2 years for 628 patients (91.5%). The baseline characteristics of the study cohort are presented in Table 1.

**TABLE 1:** Baseline characteristics.

	Missing data, *n* (%)	
Numbers, *n*		686
Age, years	0	41.8 ± 11.43
Baseline BMI, kg/m^2^	0	41.8 ± 5.74
Waist circumference, cm	13 (1.9%)	124.5 ± 13.66
Sex	0	
Women		513 (74.8%)
Men		173 (25.2%)
Comorbidities	0	
Type‐2 diabetes		87 (12.7%)
Hypertension		193 (28.1%)
Dyslipidemia		71 (10.3%)
Sleep apnea		108 (15.7%)
Depression		96 (14.0%)
Smoking	10 (1.5%)	
Active smoking		76 (11.2%)
History of smoking		203 (30.0%)
Never smoked		397 (58.7%)
Education	5 (0.7%)	
Primary school		65 (9.5%)
Secondary school		449 (52.6%)
Higher education		167 (24.5%)
FHL	9 (1.3%)	
Inadequate		113 (16.7%)
Problematic		269 (39.7%)
Sufficient		295 (43.6%)
C & C HL	24 (3.5%)	
Inadequate		42 (6.3%)
Problematic		236 (35.6%)
Sufficient		384 (58.0%)
GSE	21 (3.1%)	
Low		266 (40.0%)
High		399 (60.0%)

*Note*: Data is given as mean ± standard deviation (SD) for continuous variables and frequency (percentage) for categorical variables.

Abbreviations: BMI, body mass index; C & C HL, communicative and critical health literacy; FHL, functional health literacy; GSE, general self‐efficacy.

In this sample, 382 patients (56.4%) had limited functional health literacy (i.e. inadequate or problematic level), 278 (41.9%) had limited communicative and critical health literacy (i.e. inadequate or problematic level), and 266 (40%) reported low general self‐efficacy (Table 1).

Laparoscopic Roux‐en‐Y gastric bypass was the most common procedure (*n* = 394; 57.4%). Altogether, 292 patients (42.6%) underwent LSG. In LRYGB, the mean length of the biliopancreatic and alimentary limbs was 55 ± 8 and 112 ± 11 cm, respectively. The jejunojejunostomy was constructed using a single stapler technique in 344 patients (87.3%) and a double stapling technique in 50 patients (12.7%). Any type of staple line reinforcement during LSG was used in 119 patients (40.8%) and no reinforcement occurred in 167 (57.2%), while this information was missing for six operations (2.1%). No statistically significant difference was seen between health literacy, self‐efficacy, and surgical method (Table S1).

### 3.1 Weight‐related outcome

There were no significant differences in weight‐related outcomes at 1 and 2 years after surgery depending on the preoperative degree of health literacy and general self‐efficacy (Table 2).

**TABLE 2:** Weight‐related outcomes at 1 and 2 years after surgery.

	1 year after surgery				2 years after surgery			
	BMI‐loss (kg/m^2^)	TWL (%)	EBMIL (%)	*p*‐Value^a^	BMI‐loss (kg/m^2^)	TWL (%)	EBMIL (%)	*p*‐Value^a^
FHL								
Sufficient	12.7 ± 3.68	30.6 ± 7.59	80.9 ± 23.16	Ref	12.4 ± 4.23	29.9 ± 8.95	79.2 ± 25.94	Ref
Problematic	12.6 ± 3.87	30.0 ± 7.68	78.2 ± 22.34	.597	12.7 ± 4.68	29.8 ± 9.05	77.3 ± 24.60	.535
Inadequate	13.5 ± 4.21	31.7 ± 8.58	81.5 ± 25.39	.225	13.2 ± 4.55	30.9 ± 9.29	79.6 ± 25.19	.995
C & C HL								
Sufficient	13.0 ± 3.73	31.0 ± 7.55	80.6 ± 23.05	Ref	12.9 ± 4.38	30.8 ± 8.97	80.0 ± 25.64	Ref
Problematic	12.4 ± 3.93	29.6 ± 8.00	78.1 ± 23.25	.357	12.0 ± 4.60	28.6 ± 9.11	75.0 ± 24.66	.166
Inadequate	13.1 ± 4.72	31.0 ± 9.38	81.1 ± 23.80	.926	12.9 ± 4.68	30.7 ± 9.17	79.9 ± 22.80	.855
GSE								
High	12.6 ± 3.74	30.4 ± 7.81	80.7 ± 22.75	Ref	12.5 ± 4.29	30.0 ± 8.96	79.3 ± 24.81	Ref
Low	13.1 ± 3.98	30.8 ± 7.84	79.4 ± 24.01	.968	12.8 ± 4.74	30.0 ± 9.23	77.3 ± 26.13	.915

Abbreviations: BMI, body mass index; C & C HL, communicative and critical health literacy; EBMIL, excess BMI‐loss; FHL, functional health literacy; GSE, general self‐efficacy; TWL, total weight loss.

^a^ *p*‐Values were calculated for EBMIL, adjusted for age, sex, BMI, and operative method.

### 3.2 Obesity‐related problems

Patients with lower functional health literacy, communicative and critical health literacy, and/or general health literacy reported higher obesity‐related problems (indicating lower HRQoL) at baseline. This difference increased after surgery for patients with preoperative inadequate functional health literacy, or inadequate or problematic communicative and critical health literacy, and for those who reported low general self‐efficacy at baseline (Table 3).

**TABLE 3:** Obesity‐specific health‐related quality of life (HRQoL), measured using the Obesity Related Problem scale (OP) at 1 and 2 years after surgery.

	Baseline OP score	1 year after surgery			2 years after surgery		
		OP score	Delta OP	*p*‐Value^a^	OP score	Delta OP	*p*‐Value^a^
FHL							
Sufficient	58.0 ± 26.39	12.1 ± 16.99	44.6 ± 26.90	Ref	15.1 ± 20.24	41.7 ± 30.14	Ref
Problematic	59.5 ± 26.87	17.1 ± 21.87	43.6 ± 29.01	.112	17.9 ± 22.26	42.7 ± 28.55	.189
Inadequate	62.0 ± 27.62	22.9 ± 25.00	35.5 ± 32.32	.002	31.0 ± 27.38	27.9 ± 35.82	.008
C & C HL							
Sufficient	58.0 ± 27.42	13.2 ± 18.87	42.9 ± 29.27	Ref	15.8 ± 21.06	42.0 ± 30.95	Ref
Problematic	62.2 ± 24.88	18.6 ± 21.99	45.9 ± 27.54	.204	22.5 ± 24.18	40.0 ± 28.77	.034
Inadequate	61.2 ± 28.61	23.3 ± 26.69	30.2 ± 30.79	<.001	28.9 ± 29.56	19.7 ± 37.11	.037
GSE							
High	56.2 ± 28.40	11.7 ± 17.38	43.8 ± 29.11	Ref	14.6 ± 19.71	41.4 ± 32.36	Ref
Low	63.6 ± 23.54	21.2 ± 23.06	42.5 ± 26.95	.042	24.6 ± 25.42	38.9 ± 27.95	.005

Abbreviations: C &C HL, communicative and critical health literacy; FHL, functional health literacy; GSE, general self‐efficacy (1 = Low [<30], 2 = High [>31]).

^a^ *p*‐Values were calculated for delta OP, adjusted for age, sex, BMI, operation method, and baseline OP.

### 3.3 Health‐related quality of life

Patients with lower functional health literacy, communicative and critical health literacy, and general self‐efficacy reported lower mental as well as physical HRQoL at baseline. All groups improved in HRQoL at 1 and 2 years after surgery. A significant difference remained only for mental aspects in patients with preoperative inadequate functional health literacy at 1 year after surgery and for inadequate communicative and critical health literacy at 2 years after surgery (Table 4).

**TABLE 4:** Mental and physical health‐related quality of life (HRQoL), measured using the RAND‐36 instrument, at 1 and 2 years after surgery.

	Mental, base‐line	Mental, 1 year	*p*‐Value^a^	Mental, 2 years	*p*‐Value^a^	Physical, baseline	Physical, 1 year	*p*‐Value^a^	Physical, 2 years	*p*‐Value^a^
FHL										
Sufficient	70.4 ± 18.77	80.6 ± 14.71	Ref	74.1 ± 20.00	Ref	62.6 ± 21.10	86.0 ± 16.56	Ref	82.2 ± 20.37	Ref
Problematic	65.1 ± 20.22	77.5 ± 17.81	.356	73.1 ± 19.06	.471	59.6 ± 21.18	83.5 ± 17.53	.760	82.8 ± 16.80	.965
Inadequate	57.0 ± 21.53	71.3 ± 20.55	.045	64.6 ± 23.90	.071	53.2 ± 19.99	82.6 ± 16.11	.491	76.5 ± 22.67	.538
C & C HL										
Sufficient	68.7 ± 19.94	79.7 ± 16.64	Ref	73.9 ± 19.92	Ref	62.5 ± 20.80	86.2 ± 15.66	Ref	83.0 ± 19.07	Ref
Problematic	64.0 ± 19.94	75.6 ± 17.48	.070	71.1 ± 19.85	.261	57.5 ± 20.94	82.0 ± 18.18	.174	79.6 ± 19.7	.095
Inadequate	56.4 ± 23.34	72.9 ± 20.93	.160	60.5 ± 26.64	.013	49.0 ± 21.46	82.6 ± 15.64	.608	72.0 ± 22.51	.286
GSE										
High	69.7 ± 19.59	81.2 ± 15.02	Ref	74.7 ± 19.21	Ref	62.6 ± 21.50	86.7 ± 14.88	Ref	83.5 ± 18.23	Ref
Low	60.8 ± 20.53	72.4 ± 19.42	.092	68.7 ± 21.49	.291	56.1 ± 20.32	81.1 ± 19.15	.160	79.2 ± 20.80	.295

Abbreviations: C & C HL, communicative and critical health literacy; FHL, functional health literacy; GSE, general health literacy (1 = low [<30], 2 = high [>31]).

^a^ *p*‐Values were adjusted for age, sex, body mass index (BMI), operative method, and baseline sum scores for mental and physical health.

### 3.4 Length of stay, readmission, and complications

The median duration of hospital stay was 1 (interquartile range (IQR) 1–1) day. The proportion of patients who stayed >1 day was higher among patients with inadequate functional health literacy (risk ratio (OR) 1.76; 95% confidence interval (CI) 1.09–2.86, *p* = 0.013) and inadequate communicative and critical health literacy (RR 2.12; 95% CI 1.15–3.90, *p* = 0.019) when compared to sufficient levels. No significant difference was seen regarding general self‐efficacy. The 30‐day readmission rate was 5.4% (*n* = 37), with no significant difference related to levels of health literacy or general self‐efficacy.

Postoperative complications during the first 30 postoperative days were reported in 51 patients (7.5%), with 22 considered to be serious postoperative complications (3.2%). No significant differences were seen in complication rates with regard to levels of health literacy or general self‐efficacy (Table 5).

**TABLE 5:** Early postoperative outcomes.

	Readmission, *n* (%)	*p*‐Value^a^	Patients with longer than median length of stay, *n* (%)	*p*‐Value^a^
FHL				
Sufficient	17 (5.8%)	Ref	34 (11.5%)	Ref
Problematic	12 (4.5%)	.399	32 (11.9%)	.807
Inadequate	7 (6.3%)	.930	23 (20.4%)	.015
C &C HL				
Sufficient	23 (6.0%)	Ref	43 (11.2%)	Ref
Problematic	9 (3.9%)	.268	36 (15.3%)	.122
Inadequate	4 (9.5%)	.382	10 (23.8%)	.011
GSE				
High	24 (6.0%)	Ref	51 (12.8%)	Ref
Low	12 (4.6%)	.323	38 (14.3%)	.454

Abbreviations: C & C HL, communicative & critical health literacy; FHL, functional health literacy; GSE, general self‐efficacy.

^a^ *p*‐Values were adjusted for age, sex, body mass index (BMI), and operative method.

## 4 DISCUSSION

In this prospective cohort study, preoperative functional health literacy, communicative and critical health literacy, and general self‐efficacy were not associated with differences in weight loss at 1 or 2 years after bariatric surgery. However, despite similar weight loss, inadequate health literacy and low general self‐efficacy scores were associated with reduced quality of life in general and related to obesity‐related problems. Inadequate health literacy was also associated with prolonged length of stay.

Health literacy is receiving increasing attention and has been suggested to contribute to obesity development.^38^ For patients who have undergone bariatric surgery, everyday life includes decisions and choices about, for example, when and how to contact health care,^39^ be compliant with medication,^9^ or attend follow‐up appointments,^9^ tasks that may be more difficult for people with limited health literacy.^39^ Furthermore, lower formal education is related to health literacy level,^39^ which in turn may contribute to difficulties in postoperative recovery.^9^ Another factor that may limit adherence to postoperative health recommendations after bariatric surgery is psychiatric comorbidities, for example, attention deficit hyperactivity disorder.^40^

Data from previous studies suggests that 30%–40% of native Swedish speakers do not have sufficient health literacy.^41^, ^42^ In our study, a total of 56.4% (*n* = 382) had limited functional health literacy (i.e. inadequate or problematic levels) and 41.9% (*n* = 278) had limited communicative and critical health literacy (i.e. inadequate or problematic levels). There were no significant differences in weight loss between different levels of health literacy. While previous research about this association is inconclusive,^12^, ^13^, ^14^, ^15^ our data corroborates a previous study suggesting that health literacy may not influence the maximum weight loss after bariatric surgery.^14^ In addition, there were no significant differences between low or high general self‐efficacy and weight loss. Self‐efficacy may have a mediating role in achieving and maintaining lifestyle changes.^16^ Self‐efficacy appears to improve over time^17^, ^18^ after bariatric surgery, which may explain this lack of association. Further, personal characteristics such as health literacy and self‐efficacy may be less influential during the initial 2‐year weight loss phase.^14^

It is well known that patients with obesity report poorer HRQoL.^1^ After bariatric surgery, HRQoL scores improve,^1^, ^17^ which was also found in our study. However, poorer mental health was seen at 1 year postoperatively for those with inadequate functional health literacy, and after 2 years for those with inadequate communicative and critical health literacy. Further, those with low general self‐efficacy scores still had more obesity‐related problems, according to the OP scale. This may indicate that these patients need extra support and perhaps other interventions, which is an important issue to address in future studies.

Our results confirm previous reports,^43^ that the level of health literacy is associated with the length of hospital stay. One reason for this might be difficulties in knowing what questions to ask^39^ and in understanding medical terms.^39^ This is troublesome since medical/health communication, in its best form, should primarily be driven by informed patient preference,^39^ including individual choices and decisions about health and medical treatments.^39^ Research investigating health information reports that most health‐related materials online or in brochures are written for persons with higher reading skills.^44^, ^45^ If health information communication is to be improved and simplified, it needs to be written in plain language, separating information into clear, short sections (e.g., using bullet points), and utilising clear visual aids to increase both readability and understandability for patients with limited health literacy.^39^ Also, the teach‐back method can be used, which requires the patient to explain the information in his or her own words.^39^, ^46^ Given the paucity of research about health literacy in surgical populations^9^ and the importance of providing person‐centred care,^39^ which includes talking to patients and delivering information that is understandable,^39^ this is also an important topic to address in future investigations. Another aspect to consider is the difficulty for healthcare professionals (HCPs) in knowing whether a patient has limited or sufficient health literacy. A newly published study reports a 61.1% accuracy rate when HCPs estimate patients' health literacy levels using a validated questionnaire.^47^ This illustrates the challenge for HCPs to identify who needs extra information. To reduce the risk of inequities, healthcare should be health literacy‐friendly to ensure that everyone, regardless of their level of health literacy, can access, understand, assess, and apply health information.

Prolonged length of stay also indicates increased resource consumption. Understanding factors related to longer hospital stays after bariatric surgery can help enhance perioperative care and reduce costs.^48^

There is a lack of interventional studies aimed at improving health communication in surgical settings.^9^ However, it is aimed at addressing the needs of surgical populations through different patient education.^9^ To deliver patient‐centered and equal care for all patients, further studies are needed to increase our understanding of the influence of health literacy and self‐efficacy on postoperative recovery.

A strength of this study is that we included a large cohort of patients and had access to valid follow‐up data. Further, all instruments used have been psychometrically evaluated for this specific patient group.

However, this study is not without limitations. Our choice of using the Swedish version of the health literacy scales was based on the fact that the scales have few items, as we did not want to burden the patients with completing comprehensive questionnaires. In our results, there is a discrepancy between the respondents with limited functional literacy compared to those with limited communicative and critical health literacy. Since the latter involves a more complex ability, there should at least be a similar distribution between the categories. The reason for this discrepancy is unclear.

Health literacy can be measured with different instruments^9^ and recently, new instruments have been developed. Possibly, we would have obtained different results if we had used an instrument examining a comprehensive health literacy that includes all abilities in one instrument. However, shorter questionnaires may be more convenient to use in routine clinical practice.

The inclusion of patients was discontinued during the COVID‐19 pandemic, resulting in a longer inclusion period than anticipated. The study cohort included approximately 25% men, which is consistent with most studies on bariatric surgery in Northern Europe. Finally, the cohort consisted mainly of white Europeans, which may limit generalisability to other populations.

## 5 CONCLUSION

The results of the current study suggest that although patients with limited health literacy and self‐efficacy may achieve similar maximum weight loss post‐bariatric surgery to other patients, they still have lower postoperative HRQoL in terms of obesity‐related problems. An increased awareness of this association and patient‐centered support before and after bariatric surgery may be of benefit. Further studies are warranted to evaluate interventions to prevent inferior outcomes and prolonged lengths of stay for those with limited health literacy.

## AUTHOR CONTRIBUTIONS

Maria Jaensson (MJ): Conceptualization, methodology, validation, investigation, writing: original draft, visualization, project administration. Karuna Dahlberg (KD): Conceptualization, methodology, validation, investigation, writing: review. Ulrica Nilsson (UN): Conceptualization, methodology, investigation, writing: review. Erik Stenberg (ES): Conceptualization, methodology, validation, resources, formal analysis, investigation, writing: original draft, visualization, project administration. Johanna Österberg (JÖ): Investigation, validation, resources, writing: review. Anders Thorell (AT): Investigation, validation, resources, writing: review. Yang Cao (YC): Formal analysis, methodology, validation, writing: review.

## FUNDING INFORMATION

Maria Jaensson: This study was financed by grants from the Swedish State under the ALF agreement between the Swedish government and the county councils (OLL‐886141, OLL‐935386, and OLL‐960506); and from Örebro University (grant numbers ORU 2018/00376 and ORU 2018/2019). Erik Stenberg: This study was financed by grants from the Swedish State under the ALF agreement between the Swedish government and the county councils (OLL‐939106): The Bengt Ihre Foundation. Anders Thorell: This study also received financial support from The Erling‐Persson Foundation (Grant # 2019020).

## CONFLICT OF INTEREST STATEMENT

MJ, KD, AT, and YC have no conflicts of interest. UN: Hold shares in RAPP‐AB, a web application for secure data collection of postoperative recovery, unrelated to the present study. ES: Reimbursement for lecture from MSD and Johnson & Johnson Medical, and consultant fees (paid to institution) from Johnson & Johnson Medical. JÖ: Board member of the Swedish Register for Gallstone Surgery and ERCP (GallRiks). Board member of the Swedish Register for Hernia Surgery (SBR). Reimbursement for a lecture from Baxter and Mölnlycke, unrelated to this article.

## Supporting information


**TABLE S1.** Type of surgical procedure for the different groups stratified by health literacy and general self efficacy before surgery.

## Data Availability

Research data are not shared.
