# Supplementary material for: Impact of health literacy and general self‐efficacy on surgical outcomes 2 years after bariatric surgery
Source: Clin Obes. 2025 Mar 10;15(4):e70009. doi: 10.1111/cob.70009 (PMC12289404; doi:10.1111/cob.70009)
Supplement: Supplementary file 1 — TABLE S1. Type of surgical procedure for the different groups stratified by health literacy and general self efficacy before surgery. [file COB-15-e70009-s001.pdf]

Impact of health literacy and general self-efficacy on surgical outcomes 2 years after bariatric surgery

Maria Jaensson, Associate professor [a], Karuna Dahlberg, PhD [a], Yang Cao, Associate professor [b,c], Anders Thorell, Professor [d], Johanna Österberg, PhD [e,f] , Ulrica Nilsson, Professor [g], Erik Stenberg, Associate professor [h]

a) Faculty of Medicine and Health, School of Health Sciences, Örebro University, Örebro, Sweden.

b) Clinical Epidemiology and Biostatistics, School of Medical Sciences, Faculty of Medicine and Health, Örebro University, Örebro, Sweden.

c) Unit of Integrative Epidemiology, Institute of Environmental Medicine, Karolinska Institutet, Stockholm, Sweden

d) Department of Clinical Science, Danderyd Hospital, Karolinska Institutet, and Department of Surgery and Anesthesiology, Ersta Hospital, Stockholm, Sweden.

e) Department of Surgery, Mora Hospital, Mora, Sweden.

f) Department of Clinical Science and Education, Södersjukhuset, Karolinska Institute, Stockholm, Sweden

g) Department of Neurobiology, Care Sciences, and Society, Karolinska Institute, Stockholm, Sweden.

h) Department of Surgery, Faculty of Medicine and Health, Örebro University, Örebro, Sweden.

**Table S1.** Type of surgical procedure for the different groups stratified by health literacy and general self efficacy before surgery			
	Gastric bypass	Sleeve gastrectomy	P
**FHL**			
Sufficient	162 (55%)	133 (36%)	Ref
Problematic	154 (57%)	115 (43%)	0.577
Inadequate	72 (64%)	41(45%)	0.109
**C & C HL**			
Sufficient	219 (57%)	165 (43%)	Ref
Problematic	130 (55%)	106 (45%)	0.635
Inadequate	27 (64%)	15 (36%)	0.368
**GSE**			
High	222 (56%)	177 (44%)	Ref
Low	159 (60%)	107 (40%)	0.291

FHL = functional health literacy; C & C HL = communicative and critical health literacy; GSE = general self-efficacy
